# *N*-(2-Chloro-5-nitro­phen­yl)-*N*′-(3-chloro­propion­yl)thio­urea

**DOI:** 10.1107/S1600536813032662

**Published:** 2013-12-11

**Authors:** Bohari M. Yamin, Siti K. C. Soh, Siti Fairus M. Yusoff

**Affiliations:** a School of Chemical Sciences and Food Technology, Universiti Kebangsaan Malaysia, 43600 Bangi, Selangor, Malaysia

## Abstract

The title compound, C_10_H_9_Cl_2_N_3_O_3_S, adopts a *trans–cis* conformation with respect to the position of chloropropionyl and chloronitrobenzene groups respectively, against the thiono about their C—N bonds. The conformation is stabilized by an intra­molecular N—H⋯O hydrogen bond. In the crystal, there is a short Cl⋯Cl contact with a distance of 3.386 (13) Å.

## Related literature   

For related structures, see: Othman *et al.* (2010 ▶); Yamin *et al.* (2011 ▶); Yamin & Othman (2011 ▶); Yusof *et al.*, (2011 ▶).
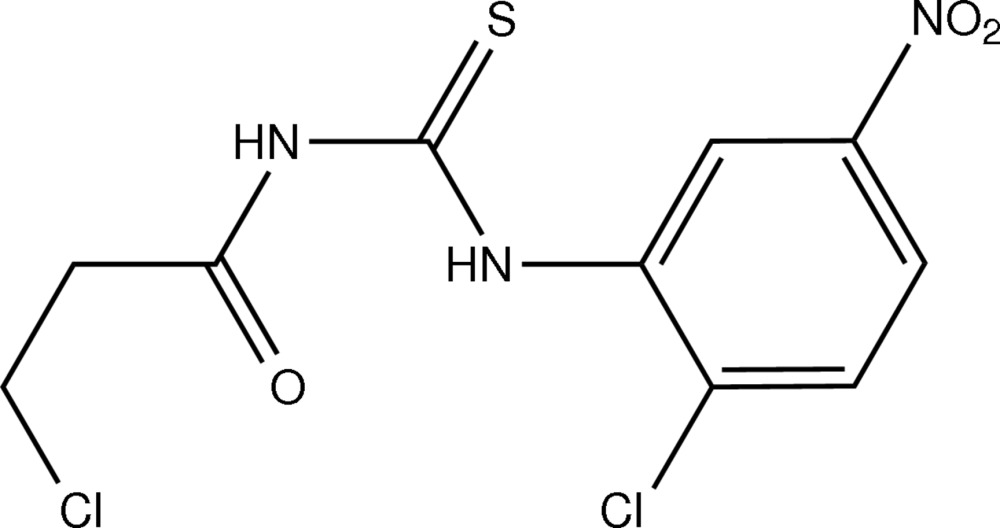


## Experimental   

### 

#### Crystal data  

C_10_H_9_Cl_2_N_3_O_3_S
*M* *_r_* = 322.16
Monoclinic, 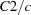

*a* = 21.764 (6) Å
*b* = 5.2284 (13) Å
*c* = 24.134 (6) Å
β = 106.388 (8)°
*V* = 2634.6 (12) Å^3^
*Z* = 8
Mo *K*α radiation
μ = 0.66 mm^−1^
*T* = 298 K
0.38 × 0.36 × 0.27 mm

#### Data collection  

Bruker SMART APEX CCD area-detector diffractometer
Absorption correction: multi-scan (*SADABS*; Bruker 2000 ▶) *T*_min_ = 0.902, *T*_max_ = 0.919
12266 measured reflections
2460 independent reflections
2116 reflections with *I* > 2σ(*I*)
*R*_int_ = 0.019

#### Refinement  

*R*[*F* ^2^ > 2σ(*F* ^2^)] = 0.036
*wR*(*F* ^2^) = 0.097
*S* = 1.05
2460 reflections
172 parameters
H-atom parameters constrained
Δρ_max_ = 0.31 e Å^−3^
Δρ_min_ = −0.23 e Å^−3^

### 

Data collection: *SMART* (Bruker, 2000 ▶); cell refinement: *SAINT* (Bruker, 2000 ▶); data reduction: *SAINT*; program(s) used to solve structure: *SHELXTL* (Sheldrick, 2008 ▶); program(s) used to refine structure: *SHELXTL*; molecular graphics: *SHELXTL*; software used to prepare material for publication: *SHELXTL* and *PLATON* (Spek, 2009 ▶).

## Supplementary Material

Crystal structure: contains datablock(s) global, I. DOI: 10.1107/S1600536813032662/fj2651sup1.cif

Structure factors: contains datablock(s) I. DOI: 10.1107/S1600536813032662/fj2651Isup2.hkl

Supporting information file. DOI: 10.1107/S1600536813032662/fj2651Isup3.cml

Additional supporting information:  crystallographic information; 3D view; checkCIF report

## Figures and Tables

**Table 1:** Hydrogen-bond geometry (Å, °)

*D*—H⋯*A*	*D*—H	H⋯*A*	*D*⋯*A*	*D*—H⋯*A*
N2—H2⋯O1	0.86	1.87	2.596 (2)	142

**Fig. 1.**
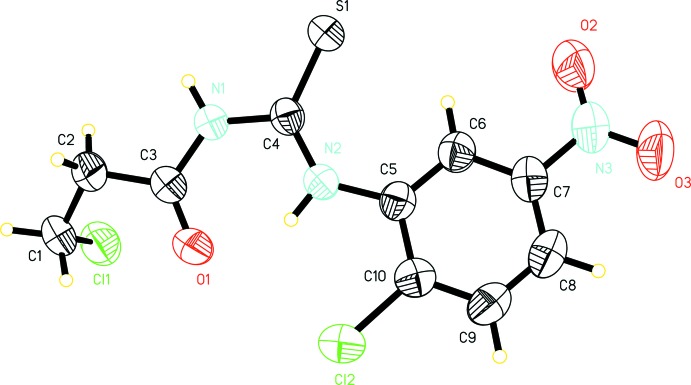
: Molecular structure of (I) with 50% probability displacement ellipsoids

**Fig. 2.**
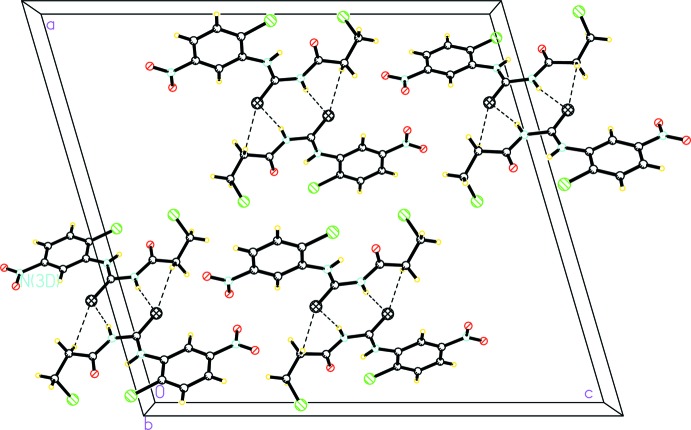
: Molecular packing of (I) in the unit cell viewed down b axis. The dashed lines indicate intermolecular hydrogen bonds.

## supplementary crystallographic information

### 1. Comment

The synthesis of halogenoalkoylthiourea will enable to further synthesized thiourea derivatives making use of the C-Cl functionality. N-(4-chlorobutanoyl)-N'-phenylthiourea (Yamin *et al.*, 2011), N-(4-chlorobutanoyl)-N'-(2-fluorophenyl)thiourea (Yusof *et al.*, 2011) and N-(4-bromobutanoyl)-N'-phenylthiourea (Yamin *et al.*, 2011) are some examples that have been reported so far. The title compound is similar to N-(3-chloropropionyl)-N'-phenylthiourea (Othman *et al.* 2010) except the presence of chlorine atom and nitro group at the ortho and meta-position of the phenyl ring, respectively.

The whole molecule is not planar (Fig. 1) because of the dihedral angle of 9.35 (8)° between benzene ring, C5-C10, and S1/O1/N1/N2/C2/C3/C4/C5/C9/C10 fragments. Both fragments are each planar with maximum deviation of 0.066 (2)Å for C10 atom from the least square plane of the benzene fragment. The molecule maintains trans-cis configuration with respect to the position of chloropropionyl and chloronitrophenyl against the thiono group about N1-C4 and N2-C4 bonds, respectively.

There is intrahydrogen bond N2-H2···O1 forming pseudo six-membered ring [N2-C4-N1-C3-O1···H2]. In the crystal packing, the molecules are linked by N1-H1···S1 intermolecular hydrogen bond (symmetry codes as in Table 1) to form centrosymmetric dimers and arranged along ac face (Fig. 2). There is also Cl2-Cl2 interaction with the contact distance of 3.386 (13) Å.

### 2. Experimental

1-chloro-4-nitrobenzene (1.57g, 0.01mol) was added into 30 ml acetone containing 3-chloropropionyl isothiocyanate (1.49g, 0.01mol). The mixture was refluxed for 2 hours. The solution was filtered and left to evaporate at room temperature. The white precipitate obtained after a few days, was washed with water and cold ethanol. The colourless crystals were obtained by recrystallization from ethanol.

### 3. Refinement

After location in the difference map, the H-atoms attached to the C and N atoms were fixed geometrically at ideal positions and allowed to ride on the parent atoms with C—H = 0.93-0.97 Å, N—H = 0.86 Å and with *U*_iso_(H)=1.2*U*_eq_(C or N).

### Crystal data

**Table:**

C_10_H_9_Cl_2_N_3_O_3_S	*Z* = 8
*M**_r_* = 322.16	*F*(000) = 1312
Monoclinic, *C*2/*c*	*D*_x_ = 1.624 Mg m^−^^3^
*a* = 21.764 (6) Å	Mo *K*α radiation, λ = 0.71073 Å
*b* = 5.2284 (13) Å	µ = 0.66 mm^−^^1^
*c* = 24.134 (6) Å	*T* = 298 K
β = 106.388 (8)°	Block, colorless
*V* = 2634.6 (12) Å^3^	0.38 × 0.36 × 0.27 mm

### Data collection

**Table:**

Bruker SMART APEX CCD area-detector diffractometer	2460 independent reflections
Radiation source: fine-focus sealed tube	2116 reflections with *I* > 2σ(*I*)
Graphite monochromator	*R*_int_ = 0.019
Detector resolution: 83.66 pixels mm^-1^	θ_max_ = 25.5°, θ_min_ = 1.8°
ω scans	*h* = −26→26
Absorption correction: multi-scan (*SADABS*; Bruker 2000)	*k* = −6→6
*T*_min_ = 0.902, *T*_max_ = 0.919	*l* = −29→29
12266 measured reflections	

### Refinement

**Table:**

Refinement on *F*^2^	Primary atom site location: structure-invariant direct methods
Least-squares matrix: full	Secondary atom site location: difference Fourier map
*R*[*F*^2^ > 2σ(*F*^2^)] = 0.036	Hydrogen site location: inferred from neighbouring sites
*wR*(*F*^2^) = 0.097	H-atom parameters constrained
*S* = 1.05	*w* = 1/[σ^2^(*F*_o_^2^) + (0.0496*P*)^2^ + 1.9444*P*] where *P* = (*F*_o_^2^ + 2*F*_c_^2^)/3
2460 reflections	(Δ/σ)_max_ < 0.001
172 parameters	Δρ_max_ = 0.31 e Å^−^^3^
0 restraints	Δρ_min_ = −0.23 e Å^−^^3^

### Special details

**Table:**

Geometry. All esds (except the esd in the dihedral angle between two l.s. planes) are estimated using the full covariance matrix. The cell esds are taken into account individually in the estimation of esds in distances, angles and torsion angles; correlations between esds in cell parameters are only used when they are defined by crystal symmetry. An approximate (isotropic) treatment of cell esds is used for estimating esds involving l.s. planes.

### Fractional atomic coordinates and isotropic or equivalent isotropic displacement parameters (Å^2^)

**Table:**

	*x*	*y*	*z*	*U*_iso_*/*U*_eq_	
Cl1	0.01680 (3)	0.29301 (14)	−0.16862 (3)	0.0761 (2)	
Cl2	0.05486 (3)	1.21922 (11)	−0.00637 (3)	0.05899 (18)	
S1	0.23748 (3)	0.45825 (11)	0.07394 (2)	0.05376 (18)	
O1	0.09544 (9)	0.7795 (4)	−0.08527 (7)	0.0804 (6)	
O2	0.20313 (12)	0.6949 (5)	0.24336 (8)	0.0984 (7)	
O3	0.15801 (11)	1.0096 (4)	0.27253 (8)	0.0953 (7)	
N1	0.17678 (8)	0.5279 (3)	−0.03529 (7)	0.0463 (4)	
H1	0.2031	0.4119	−0.0396	0.056*	
N2	0.14400 (8)	0.7943 (3)	0.02607 (7)	0.0493 (4)	
H2	0.1213	0.8542	−0.0064	0.059*	
N3	0.17078 (11)	0.8880 (5)	0.23452 (9)	0.0698 (6)	
C1	0.07531 (12)	0.4940 (5)	−0.18585 (9)	0.0621 (6)	
H1A	0.0817	0.4399	−0.2223	0.075*	
H1B	0.0599	0.6690	−0.1902	0.075*	
C2	0.13788 (11)	0.4820 (5)	−0.13939 (9)	0.0643 (6)	
H2A	0.1709	0.5636	−0.1531	0.077*	
H2B	0.1499	0.3045	−0.1310	0.077*	
C3	0.13382 (11)	0.6119 (5)	−0.08498 (9)	0.0549 (5)	
C4	0.18352 (9)	0.6053 (4)	0.02127 (8)	0.0423 (4)	
C5	0.13280 (9)	0.9128 (4)	0.07449 (8)	0.0446 (4)	
C6	0.15981 (10)	0.8404 (4)	0.13142 (9)	0.0522 (5)	
H6	0.1885	0.7046	0.1404	0.063*	
C7	0.14349 (11)	0.9724 (4)	0.17438 (9)	0.0542 (5)	
C8	0.10111 (12)	1.1733 (5)	0.16383 (11)	0.0633 (6)	
H8	0.0910	1.2581	0.1940	0.076*	
C9	0.07415 (11)	1.2451 (5)	0.10774 (11)	0.0623 (6)	
H9	0.0452	1.3802	0.0994	0.075*	
C10	0.08968 (9)	1.1184 (4)	0.06386 (9)	0.0488 (5)	

### Atomic displacement parameters (Å^2^)

**Table:**

	*U*^11^	*U*^22^	*U*^33^	*U*^12^	*U*^13^	*U*^23^
Cl1	0.0769 (4)	0.0880 (5)	0.0603 (4)	−0.0004 (3)	0.0144 (3)	0.0036 (3)
Cl2	0.0538 (3)	0.0559 (3)	0.0650 (4)	0.0055 (2)	0.0129 (3)	0.0085 (3)
S1	0.0527 (3)	0.0642 (4)	0.0402 (3)	0.0126 (2)	0.0063 (2)	0.0008 (2)
O1	0.0931 (13)	0.0910 (13)	0.0459 (9)	0.0442 (11)	0.0013 (8)	−0.0009 (9)
O2	0.1354 (19)	0.1033 (17)	0.0538 (11)	0.0309 (15)	0.0222 (11)	0.0075 (11)
O3	0.1274 (18)	0.1121 (16)	0.0543 (11)	0.0061 (13)	0.0387 (11)	−0.0152 (11)
N1	0.0480 (9)	0.0518 (10)	0.0379 (8)	0.0064 (7)	0.0101 (7)	−0.0004 (7)
N2	0.0559 (10)	0.0499 (10)	0.0389 (9)	0.0100 (8)	0.0082 (7)	0.0002 (7)
N3	0.0830 (14)	0.0789 (14)	0.0504 (11)	−0.0075 (12)	0.0233 (10)	−0.0064 (11)
C1	0.0694 (14)	0.0764 (16)	0.0370 (10)	0.0101 (12)	0.0092 (10)	0.0061 (10)
C2	0.0597 (13)	0.0895 (18)	0.0401 (11)	0.0143 (12)	0.0080 (10)	−0.0035 (11)
C3	0.0560 (12)	0.0647 (13)	0.0405 (11)	0.0111 (11)	0.0077 (9)	0.0017 (10)
C4	0.0429 (10)	0.0446 (10)	0.0386 (10)	−0.0046 (8)	0.0104 (8)	−0.0011 (8)
C5	0.0450 (10)	0.0430 (11)	0.0462 (11)	−0.0058 (8)	0.0133 (8)	−0.0055 (9)
C6	0.0551 (12)	0.0519 (12)	0.0499 (12)	−0.0007 (10)	0.0156 (9)	−0.0052 (9)
C7	0.0585 (12)	0.0588 (13)	0.0471 (11)	−0.0104 (10)	0.0177 (10)	−0.0072 (10)
C8	0.0682 (15)	0.0651 (15)	0.0640 (15)	−0.0021 (12)	0.0306 (12)	−0.0160 (12)
C9	0.0588 (13)	0.0592 (14)	0.0733 (16)	0.0069 (11)	0.0257 (12)	−0.0086 (12)
C10	0.0430 (10)	0.0474 (11)	0.0555 (12)	−0.0050 (9)	0.0128 (9)	−0.0013 (9)

### Geometric parameters (Å, º)

**Table:**

Cl1—C1	1.788 (3)	C1—H1A	0.9700
Cl2—C10	1.732 (2)	C1—H1B	0.9700
S1—C4	1.656 (2)	C2—C3	1.503 (3)
O1—C3	1.209 (3)	C2—H2A	0.9700
O2—N3	1.215 (3)	C2—H2B	0.9700
O3—N3	1.211 (3)	C5—C6	1.386 (3)
N1—C3	1.368 (3)	C5—C10	1.402 (3)
N1—C4	1.391 (2)	C6—C7	1.373 (3)
N1—H1	0.8600	C6—H6	0.9300
N2—C4	1.336 (3)	C7—C8	1.373 (3)
N2—C5	1.403 (2)	C8—C9	1.367 (3)
N2—H2	0.8600	C8—H8	0.9300
N3—C7	1.472 (3)	C9—C10	1.370 (3)
C1—C2	1.501 (3)	C9—H9	0.9300
			
C3—N1—C4	128.54 (18)	N1—C3—C2	115.24 (19)
C3—N1—H1	115.7	N2—C4—N1	114.09 (17)
C4—N1—H1	115.7	N2—C4—S1	127.65 (15)
C4—N2—C5	131.77 (17)	N1—C4—S1	118.27 (15)
C4—N2—H2	114.1	C6—C5—C10	117.74 (19)
C5—N2—H2	114.1	C6—C5—N2	125.45 (19)
O3—N3—O2	123.3 (2)	C10—C5—N2	116.79 (18)
O3—N3—C7	118.3 (2)	C7—C6—C5	118.9 (2)
O2—N3—C7	118.3 (2)	C7—C6—H6	120.5
C2—C1—Cl1	110.94 (17)	C5—C6—H6	120.5
C2—C1—H1A	109.5	C6—C7—C8	123.2 (2)
Cl1—C1—H1A	109.5	C6—C7—N3	118.4 (2)
C2—C1—H1B	109.5	C8—C7—N3	118.4 (2)
Cl1—C1—H1B	109.5	C9—C8—C7	118.1 (2)
H1A—C1—H1B	108.0	C9—C8—H8	120.9
C1—C2—C3	111.67 (19)	C7—C8—H8	120.9
C1—C2—H2A	109.3	C8—C9—C10	120.1 (2)
C3—C2—H2A	109.3	C8—C9—H9	119.9
C1—C2—H2B	109.3	C10—C9—H9	119.9
C3—C2—H2B	109.3	C9—C10—C5	121.8 (2)
H2A—C2—H2B	107.9	C9—C10—Cl2	118.31 (18)
O1—C3—N1	122.5 (2)	C5—C10—Cl2	119.85 (16)
O1—C3—C2	122.22 (19)		
			
Cl1—C1—C2—C3	−70.4 (3)	C5—C6—C7—N3	177.91 (19)
C4—N1—C3—O1	2.2 (4)	O3—N3—C7—C6	177.6 (2)
C4—N1—C3—C2	−178.1 (2)	O2—N3—C7—C6	−4.9 (3)
C1—C2—C3—O1	−25.3 (4)	O3—N3—C7—C8	−4.8 (3)
C1—C2—C3—N1	155.0 (2)	O2—N3—C7—C8	172.7 (2)
C5—N2—C4—N1	175.19 (19)	C6—C7—C8—C9	−0.2 (4)
C5—N2—C4—S1	−4.5 (3)	N3—C7—C8—C9	−177.7 (2)
C3—N1—C4—N2	−3.1 (3)	C7—C8—C9—C10	−0.2 (4)
C3—N1—C4—S1	176.60 (18)	C8—C9—C10—C5	0.4 (3)
C4—N2—C5—C6	−4.7 (3)	C8—C9—C10—Cl2	−179.30 (18)
C4—N2—C5—C10	176.6 (2)	C6—C5—C10—C9	−0.2 (3)
C10—C5—C6—C7	−0.2 (3)	N2—C5—C10—C9	178.68 (19)
N2—C5—C6—C7	−178.95 (19)	C6—C5—C10—Cl2	179.49 (15)
C5—C6—C7—C8	0.4 (3)	N2—C5—C10—Cl2	−1.7 (2)

### Hydrogen-bond geometry (Å, º)

**Table:**

*D*—H···*A*	*D*—H	H···*A*	*D*···*A*	*D*—H···*A*
N2—H2···O1	0.86	1.87	2.596 (2)	142
